# Assessing the feasibility of fly based surveillance of wildlife infectious diseases

**DOI:** 10.1038/srep37952

**Published:** 2016-11-30

**Authors:** Constanze Hoffmann, Melanie Stockhausen, Kevin Merkel, Sébastien Calvignac-Spencer, Fabian H. Leendertz

**Affiliations:** 1 Epidemiology of highly pathogenic microorganisms, Robert Koch-Institute, Berlin, Germany; 2 Albert-Ludwigs-University, Faculty of Biology, Freiburg, Germany

## Abstract

Monitoring wildlife infectious agents requires acquiring samples suitable for analyses, which is often logistically demanding. A possible alternative to invasive or non-invasive sampling of wild-living vertebrates is the use of vertebrate material contained in invertebrates feeding on them, their feces, or their remains. Carrion flies have been shown to contain vertebrate DNA; here we investigate whether they might also be suitable for wildlife pathogen detection. We collected 498 flies in Taï National Park, Côte d’Ivoire, a tropical rainforest and examined them for adenoviruses (family *Adenoviridae*), whose DNA is frequently shed in feces of local mammals. Adenoviral DNA was detected in 6/142 mammal-positive flies. Phylogenetic analyses revealed that five of these sequences were closely related to sequences obtained from local non-human primates, while the sixth sequence was closely related to a murine adenovirus. Next-generation sequencing-based DNA-profiling of the meals of the respective flies identified putative hosts that were a good fit to those suggested by adenoviral sequence affinities. We conclude that, while characterizing the genetic diversity of wildlife infectious agents through fly-based monitoring may not be cost-efficient, this method could probably be used to detect the genetic material of wildlife infectious agents causing wildlife mass mortality in pristine areas.

The recent epidemic of Ebola virus in West Africa has again shown the relevance of emerging infectious diseases (EID) for global public health and economies^1^,^2^. Like the Ebola virus, most EIDs are of zoonotic origin and involve wildlife reservoir hosts^3^. Emergence is likely facilitated by increased contact of humans and domestic animals with wildlife combined with insufficient access to health care^4^. Therefore, it is most intense in resource-poor tropical regions, such as those in Central and West Africa where the Ebola virus repeatedly emerged^3^,^5^. To prevent disease emergence, monitoring of wildlife infectious agents in these hotspot regions is essential. Some prior knowledge on these microorganisms’ genetic diversity may accelerate the identification of early-stage spillover events and help mitigate their outcome.

Surveillance of wildlife infectious agents faces many hurdles. Poor infrastructure and limited resources in remote areas hinder sample collection, storage and transport in such a way that setting up the logistic framework necessary for acquiring wildlife samples is often challenging. This is even more complicated when endangered species are to be monitored. For instance, non-human primate species, which are promising candidates for detecting potential zoonotic agents^6^, are generally strictly protected.

This means that only non-invasive sampling methods can be used, e.g. fecal sampling^7^. Fortunately, nucleic acids of vertebrate-infecting microorganisms exhibiting a variety of tissue tropisms have already been recovered from fecal samples^8^,^9^,^10^. These methods come with some disadvantages: they incur additional organizational costs, e.g. extra staff often needs to be recruited and specifically trained, they are labor-intensive and they exclude candidate reservoir species for which fecal sampling is impractical or impossible, e.g. wild rodents. This highlights the need for the development of complementary and cost-efficient monitoring techniques.

Blood-sucking invertebrates might represent interesting alternative sources of vertebrate-pathogen nucleic acids. Blood meals of many hematophagous arthropods have been demonstrated to contain DNA from their vertebrate hosts and the pathogens for which they act as vectors, i.e. malaria in birds^11^. Besides vector-borne pathogens, it is also possible that non-vector-borne pathogens could be contained in blood meals. Interesting examples include the detection of RNA from H5N1 avian influenza A viruses in a mosquitoes’ blood meal^12^,^13^,^14^. Grubaugh and colleagues went a step further and proposed to use blood meal analysis as a tool to survey human pathogens in remote tropical locales, which they refer to as *xenosurveillance*^15^. Blood-sucking arthropods however often exhibit strong host preferences, which may be suboptimal when the objective is to survey infectious agent diversity in complex ecosystems with high biodiversity.

Non blood-sucking invertebrates feeding on vertebrate fecal matter and/or carrion, such as blow and flesh flies (here referred to simply as flies), might also be suitable for the surveillance of wildlife infectious agents. Flies are abundant and ubiquitous, have little host preference and are easy to trap^16^,^17^. We also recently showed that flies often contain DNA fragments of their mammalian hosts^16^,^17^. Finally, the genetic material of a number of hitchhiked microorganisms was already detected in flies, including food borne bacteria, e.g. *Salmonella* spp., and enteric viruses^18^,^19^,^20^,^21^. For example, Newcastle disease virus (NDV) RNA was detected in, and even virions were isolated from, flies collected in the vicinity of infected chickens. Similarly, H5N1 RNA was found in flies collected in the surroundings of a poultry farm with infected birds^22^,^23^.

These studies however focused on flies caught near high-density vertebrate populations, which raises questions about the broad applicability of this method. In this study, we investigate whether flies are suitable for vertebrate-infecting microorganism surveillance in complex ecosystems with high species richness. We analyzed flies collected in a remote tropical rainforest, Taï National Park (TNP), Côte d’Ivoire, and focused on an *a priori* favorable target: adenoviruses (AdV; family *Adenoviridae*). AdV are shed massively in feces, are usually host-specific and have already been detected in many vertebrates in TNP^24^,^25^.

## Results

Out of 498 flies, 156 (31%) contained mammalian 16 S mt DNA. We considered all mammal positive flies as suitable for AdV screening, but due to shortage of material we could only test 142 of these flies. From eight flies AdV DNA could be amplified and sequenced once and six AdV sequences could be confirmed a second time. A BLAST search revealed that four of the six sequences were ≥98% identical to a simian AdV sequence determined from a mona monkey (*Cercopithecus campbelli*, KP274048) in Côte d’Ivoire (Fly 92, Fly 101, Fly 740, Fly 1355)^26^. The remaining two sequences (Fly 381, Fly 1375) showed 100% identity with simian AdV sequences obtained from captive chimpanzees in the US (FJ025905, FJ025926) and from a wild chimpanzee in TNP (JN163974) and 98% identity with a murine AdV 2 sequence (NC014899), respectively (Table 1).

**Figure 1:**
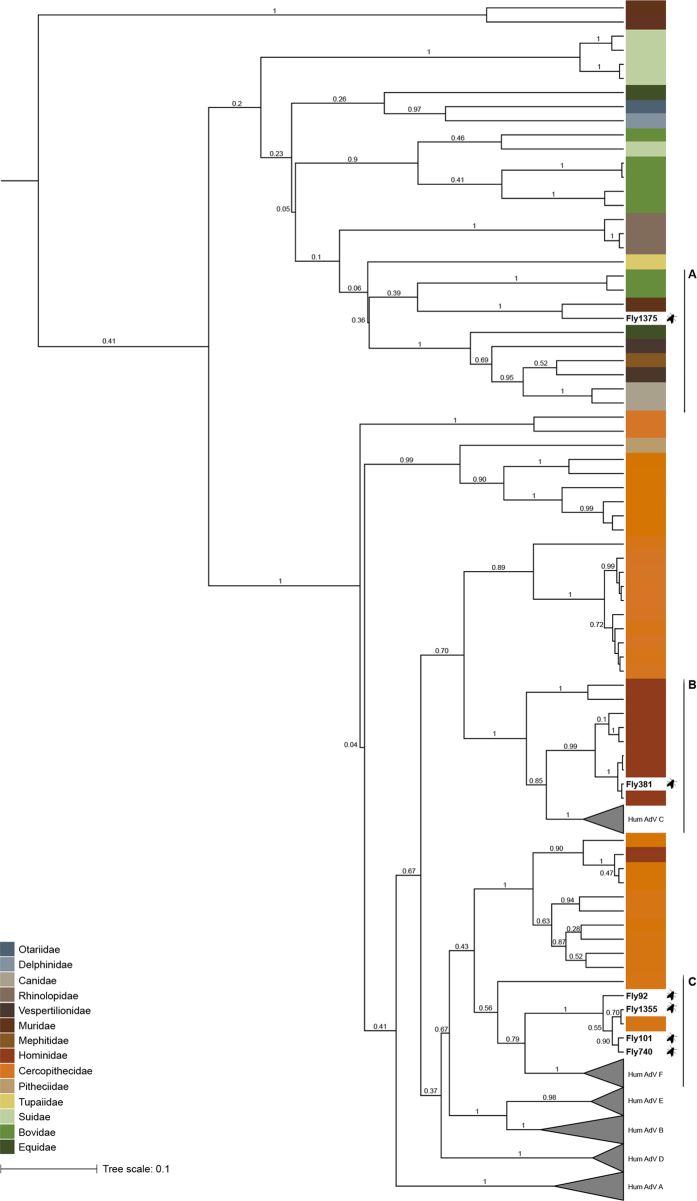
Maximum clade credibility (MCC) tree of mastadenoviruses. This MCC tree is based on the Bayesian analysis of a 370 bp long alignment of hexon gene sequences. Posterior probabilities are plotted above branches. The tree was built under a clock model and thus is rooted. Enlarged section A, B and C are shown in Fig. 2.

**Figure 2:**
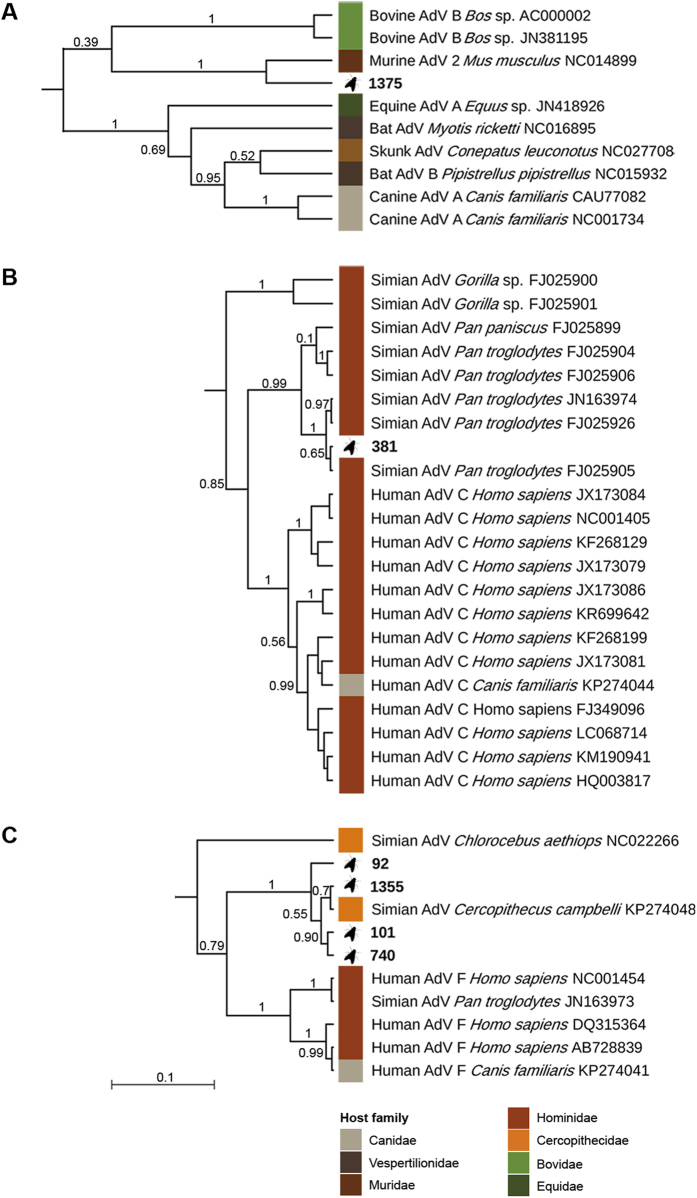
Enlarged sections of the maximum clade credibility of mastadenoviruses. The original MCC tree (Fig. 1) was based on the Bayesian analysis of a 370 bp long alignment of hexon gene sequences. Posterior probabilities are plotted above branches. Study sequences are in bold, reference sequences are represented by host name and accession number.

We also performed phylogenetic analyses in both maximum likelihood and Bayesian frameworks to better determine the position of fly-derived AdV sequences within the AdV family tree (Fig. 1). In line with the BLAST search, sequences from Fly 92, Fly 101, Fly 740 and Fly 1355 formed a well-supported clade with the mona monkey AdV sequence (aLRT 0.99, pp 1; Fig. 2C). The sequence of Fly 381 clustered with AdV sequences from captive and wild chimpanzees (FJ025905, FJ025926, JN163974, FJ025906, FJ025904, FJ0295899; aLRT 0.98, pp 1; Fig. 2B)^24^,^27^. These sequences nested within the clade corresponding to species *Human mastadenovirus C*, albeit with a much lower statistical support (HAdV-C; aLRT 0.93, pp 0.85). The sequence of Fly 1375 was most closely related to the murine AdV B (Murine AdV 2) sequence (aLRT 0.99, pp 1; Fig. 2A).

Meal analyses based on Sanger sequencing identified plausible hosts in 3 of the 6 AdV positive flies (Fly 92, Fly 101, Fly 1355; Table 1). To further investigate potential hosts, we performed an in-depth fly meal analysis of all six AdV positive flies using a metabarcoding approach. After quality trimming, 65,552 reads–8,683 to 12,278 reads per fly - were used for taxonomic assignment. Overall, we identified hosts from 9 mammal families and 10 genera/species. Five flies contained DNA from multiple hosts (Fly 92, Fly 101, Fly 381, Fly 1355, Fly 1375; Table 1). The 3 plausible hosts identified by Sanger sequencing were confirmed by this approach. Fly 740, which harbored one of the simian AdV sequences, only contained rodent DNA fragments. For the last two flies, the metabarcoding approach revealed the presence of DNA fragments belonging to plausible hosts, i.e. rodents in Fly 1375 and a hominid in Fly 381. As the hominid family contains the two closely related genera *Homo* and *Pan,* we manually checked the according sequences and were able to refine the assignment to *Pan troglodytes*.

## Discussion

We investigated the feasibility of using DNA derived from flies for the surveillance of wildlife infectious diseases. We were able to detect short AdV sequences in 6 flies, that is 4.2% of all mammal positive flies. We used these sequences for phylogenetic analyses and found that most represented AdVs known to infect monkeys and great apes in the region^24^,^25^. The close relationship of four sequences with an AdV sequence obtained from a single mona monkey supports the notion that this AdV may be relatively abundant in the region^26^. The fifth fly-based simian AdV sequence clustered with HAdV-C sequences and clearly belonged to the chimpanzee clade. HAdV-C viruses are very host-specific and seem to have co-diverged with their hominid hosts^25^. We also detected what is likely a new rodent AdV, thereby underlining the potential of flies to also monitor small-bodied species. Finally, our high-throughput fly meal analyses identified multiple hosts, including plausible ones, in 5 of 6 AdV positive flies. These results demonstrate that fly-based analyses allow for the simultaneous characterization of microorganism genetic diversity and their distribution in local mammalian hosts.

In comparison with detection rates in fecal samples (11 to 58%), the AdV detection rate in flies appears low^25^,^28^. This might result from the extreme dilution of vertebrate-infecting microorganisms in carrion flies, which itself results from the interplay of meal quantity, quality, frequency and the speed of digestive processes^29^. Given this low detection rate, systematic screenings would probably only make sense where fly collections established for other purposes, e.g. mammal diversity assessment, are available. Sample pooling combined with deep sequencing of PCR products may help decrease the workload and costs of such a screening approach.

Further investigations are needed to determine the extent to which the approach described here is applicable to other microorganisms. The low detection rate of AdV sequences in flies suggests that surveillance of non-enteric microorganisms might be complicated. However, in the case of outbreaks with massive production of microorganisms, e.g. Ebola virus outbreaks^30^, there might be a good chance that pathogen nucleic acids are detectable in flies. Of course, the detection probability will depend on the biology of the microorganism of interest. Here also, high throughput sequencing approaches (including shotgun sequencing) could open up new perspectives, as recently shown with mosquitoes^13^,^14^. It was recently demonstrated that portable sequencing devices such as the MinION (Oxford Nanopore Technologies, Oxford, United Kingdom) can be used for on-site sequencing in outbreak situations^31^,^32^. These technologies only require a basic molecular laboratory in the field. Such laboratories currently allow users to perform sequencing, though the high error rate and relatively low throughput of MinIONs currently limit them to amplicon sequencing based approaches. The limited needs in the present study suggest it could be feasible to conduct fly/amplicon-based wildlife surveillance during major outbreaks, including in resource-poor countries.

Invertebrates other than carrion flies and blood sucking arthropods might also constitute a valuable source of information on vertebrate-infecting microorganisms. For example, leeches can ingest several times their weight host blood in a single meal and could therefore be seen as long-term blood tanks. Most recently, a number of viruses (with DNA or RNA genomes) were shown to persist up to four months in experimentally fed aquatic leeches, with bovine parvovirus being detectable for up to six months^33^. Terrestrial leeches, whose lifestyle might be more compatible with broad, undirected wildlife molecular epidemiology, were also recently shown to allow retrieval of their host’s DNA^34^. Both aquatic and terrestrial leeches warrant a careful examination of their potential as tools for wildlife microorganism sampling.

Finally, an alternative to microorganism nucleic acid detection might be the detection of antibodies reactive to these microorganisms. If this is feasible, it would open the potential to examine wildlife exposure to microorganisms. Detection of trypanosome-reactive antibodies from a number of haematophagous dipterans was reported as early as 1962 35. This potential tool then fell into a long-lasting oblivion until its recent rediscovery by Barbazan and colleagues, who showed that blood-fed mosquitoes contain detectable levels of various virus-reactive antibodies^36^. Determining whether invertebrate-based serological surveys can be conducted in the wild promises to be an exciting area of future research.

## Material and Methods

### Sample collection

Sample collection was performed with the permission of the Ivorian national parks authorities (OIPR) and the ministry of research of Côte d’Ivoire. Flies used in this study were captured in Taï National Park, Côte d’Ivoire, a tropical rainforest with remarkable mammal biodiversity. Overall, 498 flies were captured using customized fly traps consisting of a pyramidal mosquito net over a plastic bowl containing a commercial bait (Unkonventionelle Produkte Feldner, Waldsee, Germany) or a piece of meat^16^. After collection, flies were either placed in Cryotubes (Thermofischer, Waltham, MA, USA), and stored in liquid nitrogen tanks, or in 50 ml Falcon tubes (Carl Roth, Karlsruhe, Germany) containing silica and stored either at ambient temperature or 4 °C.

### Nucleic acid extraction

Single flies were cut with sterile scissors and crushed in 250 μL phosphate buffer saline using the FastPrep^®^-24 Instrument (MP Biomedicals, Illkirch, France). One hundred μL of the resulting mix were then used to extract carrion fly DNA using the EURx GeneMatrix Stool DNA Purification Kit (Roboklon, Berlin, Germany).

### Identification of flies suitable for adenovirus screening

We considered that flies containing mammal DNA would be suitable for adenovirus screening. To show the presence of mammal DNA, we first ran a real time PCR targeting a short 130 bp fragment of mitochondrial 16 S rRNA (16Smam1 5′-CggTTggggTgACCTCggA-3′ and 16Smam2 5′-gCTgTTATCCCTAgggTAACT-3′)^37^. Reaction volume was 12.5 μL and contained 6.25 μL GoTaq^®^ qPCR Master Mix (Promega, Fitchburg, WI, USA), 0.2 μM of each primer, 1 μM of blocking primer (human blocking primer 16Smam_blkhum3 5′-CggTTggggCgACCTCggAgCAgAACCC—spacerC3^38^), 1 μL DNA extract or 1 μL of a diluted African palm civet (*Nandinia binotata*) PCR product (10^1^–10^5^ molecules per μL) for standard reactions. Cycling conditions were: 95 °C 5 min, 40 cycles [95 °C 15 sec, 64 °C 1 min], and 95 °C 1 min, 55 °C 30 sec, 95 °C 30 sec. For all positive flies, we attempted to generate PCR products for sequencing using the same amplification primer pair as for the real-time PCR^16^,^37^. PCRs were carried out in a total volume of 25 μL and seeded with 200 ng of DNA (DNA concentration > 40 ng/μL) or 5 μL DNA (DNA concentration <40 ng/μL). Reactions contained 0.2 mM dNTP (with dUTP replacing dTTP), 4 mM MgCl_2_, 0.2 μM of each primer, 1 μM of two different blocking primer (human blocking primer 16Smam_blkhum3 5′-CggTTggggCgACCTCggAgCAgAACCC—spacerC3^38^, pig blocking primer 16Smam_blksus1 5′-CggTTggggTgACCTCggAgTACAAAAAAC—spacerC3^16^), 0.3 U Amperase^®^ uracil N-glycolsylase (Invitrogen), 1.25U Platinum^®^ Taq Polymerase (Invitrogen) and 2.5 μL 10x PCR Buffer (Invitrogen). Cycling condition were: 45 °C 7 min, 95 °C 15 min, 42 cycles [95 °C 30 sec, 64 °C 30 sec, 72 °C 1 min], and 72 °C 10 min. PCR products were cleaned up with ExoSAP-IT^®^ (Affymetrix, Santa Clara, CA, USA) and sequenced in both directions according to Sanger’s method using the BigDye Terminator kit v3.1 (Thermofischer). All chromatograms were evaluated using the software Geneious Pro v9.1.3 (Biomatters Ltd., Auckland, New Zealand)^39^. Sequences were assigned to species or higher taxa using BLAST^40^ and following the rationale depicted in Calvignac-Spencer and colleague’s study^16^. Most of these assignments were made in course of the study of Schubert & Stockhausen *et al*. 2014. Flies that produced a band of the expected size but did not yield interpretable sequences were also used for AdV screening.

### Adenovirus screening

We implemented various countermeasures to minimize contamination. To avoid cross-contamination with native AdV DNA, DNA extraction was never performed simultaneously with other sample types (fecal and tissue samples). To minimize contamination with PCR products, PCR setup and post-PCR analysis steps were performed in separate, dedicated rooms. In addition, a glovebox exclusively dedicated to fly analysis was used to set up all PCR performed for this study. We also used dUTP instead of dTTP and cleaned our confirmatory reactions with uracil n-glycosylase (UNG) to further reduce the likelihood of carry over contamination with PCR products (see below). It should also be noted that before this study, AdV sequences had never been amplified in the laboratory where AdV screening was performed.

A semi-nested PCR system described by Pauly *et al*.^26^ was used for detection of adenoviruses^26^. Primers had been designed for the generic detection of mastadenoviruses and targeted a short 160 bp fragment of the hexon gene (6500 s 5′CgCAgTggKCNTWCATgCACAT-3′, 6500 s 5′-ACCCACgAYgTSACNACNgA-3′, 6500as 5′-gTgCCggTgTANggYTTRAA-3′). All PCRs were carried out in a 25 μL mix containing 0.2 mM dNTP (with dUTPs replacing dTTPs), 4 mM MgCl_2_, 0.2 μM of each primer, 1.25U Platinum^®^ Taq Polymerase (Invitrogen) and 2.5 μL 10x PCR Buffer (Invitrogen). Reactions of the first round were seeded with 200 ng DNA extract or 5 μL if DNA concentration was below 40 ng/μL and the second round with 1.5 μL of 1:40 diluted PCR product of the first round. Cycling conditions were: 95 °C 5 min, 40 cycles [95 °C 30 sec, 56 °C 30 sec, 72 °C 60 sec], and 72 °C 10 min. PCR products were Sanger sequenced as described above. Flies apparently containing amplifiable AdV nucleic acids were confirmed using the same assay but including 0.3 U Amperase^®^ UNG (Invitrogen) in the first round reaction so as to minimize the risk of contamination with PCR products. Again, PCR products that yielded a band were sequenced using the Sanger method. All chromatograms were evaluated using Geneious Pro v9.1.3 39 and the respective sequences were confirmed to be adenoviral by a BLAST search^40^.

### Phylogenetic analysis

The dataset used for phylogenetic analysis comprised AdV sequences generated in this study (n = 6) and hexon gene sequences extracted from all available complete genomes of the genera *Mastadenovirus* and *Atadenovirus* (n = 506). This set of sequences was reduced to only contain unique sequences using FaBox v1.41 41. All remaining 363 sequences were aligned at the nucleotide level in SeaView v4 42, using the MUSCLE algorithm^43^. Conserved blocks were selected using Gblocks^44^ (as implemented in SeaView v4) resulting in an alignment of 370 positions. After block selection, sequences were de-replicated again using FaBox v1.41 (218 unique sequences). The best-fit model of nucleotide substitution was selected using JModelTest v2.14 and the Bayesian information criterion^45^ (SYM + I + G). Maximum likelihood (ML) as well as Bayesian frameworks were used for tree reconstruciton. The ML tree was reconstructed using PhyML v3.0 46. Branch support was estimated using SH-like approximate likelihood-ratio tests. The Bayesian phylogeny was estimated using BEAST v1.8.2 47 under the assumption of a relaxed clock (lognormal) and a Yule tree prior. Multiple Markov chain Monte Carlo analyses were run; convergence and effective sample sizes were checked in Tracer v1.6 (combined effective sampling size was >200). Tree files of all runs were combined using LogCombiner v1.8.2 and the maximum clade credibility tree was extracted using TreeAnnotator v1.8.2.

### Identification of fly meals

We performed in depth meal analysis of the 6 AdV-positive flies using a metabarcoding approach. Primary 16 S amplicons were generated with the same primers and under the same conditions as mentioned above^16^,^37^. Preparation of the generated amplicons for the Illumina MiSeq (San Diego, CA, US) sequencing platform included a first PCR in which Illumina specific overhang adapters are added to the fragment and a second PCR in which sequencing adapters and sample specific indexes were added. The first PCR reaction contained 25 μL of 1:50 diluted PCR-product, 0.2 mM dNTP, 4 mM MgCl_2_, 0.2 μM of each fusion primer (16Smam primer plus Illumina specific adapter sequence), 1 μM of each blocking primer, 1.25 U Platinum^®^ Taq Polymerase (Invitrogen) and 10x PCR Buffer (Invitrogen) in a total volume of 50 μL. Cycling conditions were: 95 °C 5 min, 5 cycles [95 °C 30 sec, 64 °C 30 sec, 72 °C 1 min], and 72 °C 10 min. The second PCR mix contained 5 μL of cleaned PCR product from the first PCR, 25 μL KAPA HiFiTM Hot Start ReadyMix (PeqLab, Erlangen, Germany), 10 μM of each Nextera Index Primer (Illumina) in a total volume of 50 μL. Cycling conditions were 95 °C 3 min, 15 cycles of 95 °C 30 sec, 55 °C 30 sec, 72 °C 30 sec followed by elongation at 72 °C for 5 min. Both the first and second PCR products were cleaned using the Agencourt^®^ AMPure^®^ XP PCR1 Purification system (Beckman Coulter, CA, USA). The final dual-indexed amplicon libraries were quantified using Quant-iT™ dsDNA Broad-Range Assay Kit (Invitrogen) and pooled equimolarly. The pool was sequenced on an Illumina MiSeq platform using the MiSeq Reagent Kit v2 (2 × 150 bp; Illumina). Raw reads were analyzed using a custom bioinformatic pipeline: paired-end reads were first merged with the program illuminapairedend of the software package OBITools v1.1.18 setting the minimum alignment score to 40. Primer sequences were then removed using the program Cutadapt v1.2.1^48^,^49^ before quality trimming was conducted with the program Trimmomatic v0.35 50 setting the quality score to 30 over a sliding window of four bases. We then de-replicated identical sequences and filtered out those that occurred less than 10 times using the obiuniq and obigrep commands of OBITools. For taxonomic assignment a reference database was built by performing an *in silico* PCR on all mammalian and vertebrate sequences available at Genbank (http://www.ncbi.nlm.nih.gov/genbank/) using the program ecoPCR v0.2 51,52. This not only contained the reference sequences themselves, but also a unique *taxid* that links each sequence to a taxonomy database where the taxonomic information was stored. For the assignment itself we used the ecotag command of OBITools. Ecotag uses the global alignment algorithm Needleman-Wunsch to find the most similar sequence to the query sequence in the reference database with a minimum identity level of 0.95 (primary reference sequence). The query sequence is then assigned to the most common recent ancestor of the primary reference sequence and the most similar reference sequence to the primary reference (secondary reference sequence).

## Additional Information

**Accession codes:** AdV sequences and raw reads of fly meal analysis are available at the European Nucleotide Archive under the accession numbers (Fly 92: LT617635; Fly 101: LT617636; Fly 381: LT617637; Fly 740: LT617638; Fly 1355: LT617639; Fly 1375: LT617640) and study accession number PRJEB14554 (sample accession numbers ERS1272921- ERS1272926).

**How to cite this article**: Hoffmann, C. *et al*. Assessing the feasibility of fly based surveillance of wildlife infectious diseases. *Sci. Rep.* **6**, 37952; doi: 10.1038/srep37952 (2016).

**Publisher's note:** Springer Nature remains neutral with regard to jurisdictional claims in published maps and institutional affiliations.

## Figures and Tables

**Table 1:** Results of AdV screening and in depth fly meal analysis.

AdV Fly ID	closest AdV BLAST hit (identity/host/accession)	Mammal 16 S - fly meal analysis			
		Sanger		NGS MiSeq	
		closest BLAST hit	species assignment (assigned raw reads)	genus assignment (assigned raw reads)	family assignment (assigned raw reads)
92	Simian AdV (98%/*Cercopithecus campbelli*/KP274048)	*Cercopithecus nictitans* 96%	*Cercopithecus nictitans* (9289)	*Cercopithecus* (9859)	Cercopithecidae (11194)
			*Colobus guereza* (1335)	*Colobus* (1335)	Suidae (123)
			*Cercopithecus campbelli* (559)	*Sus* (123)	not assigned (38)
			*Sus scrofa* (123)	not assigned (38)	
			not assigned (49)		
101	Simian AdV (99%/*Cercopithecus campbelli*/KP274048)	*Cercopithecus diana* 90%	*Cercopithecus diana* (9327)	*Cercopithecus* (10266)	Cercopithecidae (10266)
			*Cercopithecus campbell*i (825)	*Sus* (395)	Suidae (395)
			*Sus scrofa* (352)	not assigned (24)	not assigned (24)
			not assigned (181)		
381	Simian AdV (100%/*Pan troglodytes*/JN163974, FJ025905, FJ025926)	dirty sequence	not assigned (10526)	*Felis* (6657)	Felidae (6657)
			*Felis catus* (178)	not assigned (4047)	Hominidae^2^ (4047)
740	Simian AdV (98%/*Cercopithecus campbelli*/KP274048)	unknown rodent 100%^1^	*Cricetomys* sp. 1 PG-2014 (6767)	*Cricetomys* (7050)	Nesomyidae (7050)
			not assigned (5511)	not assigned (5228)	not assigned (5228)
1355	Simian AdV (100%/*Cercopithecus campbelli*/KP274048)	*Cercopithecus campbelli* 99%	*Cercopithecus campbelli* (7529)	*Cercopithecus* (7571)	Cercopithecidae (11805)
			not assigned (4318)	not assigned (4259)	not assigned (42)
				*Cercocebus* (17)	
1375	Murine AdV 2 (98%/*Mus musculus*/NC014899)	Cephalophini 97%	not assigned (5252)	not assigned (3196)	Cercopithecidae (3139)
			*Civettictis civetta* (2598)	*Civettictis* (2598)	Viverridae (2598)
			*Rattus rattus* (620)	*Cephalophus* (1916)	Bovidae (1916)
			*Neoromicia somalicus* (213)	*Rattus* (760)	Muridae (760)
				*Neoromicia* (213)	Vespertilionidae (213)
					not assigned (57)

^1^Assignment based on a local database.

^2^Manual BLAST search revealed 100% identity with *Pan troglodytes*.
